# Crystal structure of *N*′-[(*E*)-3,5-di­chloro-2-hy­droxy­benzyl­idene]-4-nitro­benzo­hydrazide di­methyl­formamide monosolvate

**DOI:** 10.1107/S2056989015018290

**Published:** 2015-10-07

**Authors:** Bibitha Joseph, N. R. Sajitha, M. Sithambaresan, E. B. Seena, M. R. Prathapachandra Kurup

**Affiliations:** a Department of Applied Chemistry, Cochin University of Science and Technology, Kochi 682 022, India; b Department of Chemistry, Faculty of Science, Eastern University, Chenkalady, Sri Lanka; c Department of Chemistry, TMJM Govt. College, Manimalakkunnu, India

**Keywords:** crystal structure, aroyl hydrazone, hydrogen bonding

## Abstract

In the title compound, C_14_H_9_Cl_2_N_3_O_4_·C_3_H_7_NO, the hydrazone mol­ecule adopts an *E* conformation with respect to azomethine bond, and the dihedral angle between the two aromatic rings [8.96 (11)°] shows that the rings are almost co-planar. The planar conformation of the mol­ecule is stabilized by the intra­molecular O—H⋯N hydrogen bond involving the OH group and azomethine N atom. The azomethine and keto bond distances [1.269 (2) and 1.210 (2) Å, respectively] are very close to the formal C=N and C=O bond lengths. The di­methyl­formamide solvent mol­ecule is connected to the hydrazone NH group *via* an N—H⋯O hydrogen bond. In the crystal, non-classical C—H⋯O and C—H⋯Cl hydrogen bonds link the mol­ecules into chains along [322]. A supra­molecular three-dimensional architecture is created by weak C—Cl⋯π [4.163 (3) Å, 83.26 (9)°] and π–π [centroid–centroid distance = 4.0395 (14) Å] inter­actions.

## Related literature   

For applications of hydrazones in supra­molecular chemistry, see: Su & Aprahamian (2014 ▸). For biological applications of hydrazones and derivatives, see: Nair *et al.* (2014 ▸); Prasanna & Kumar (2013 ▸); Holló *et al.* (2014 ▸). For the synthesis of related compounds, see: Bessy *et al.* (2006 ▸).
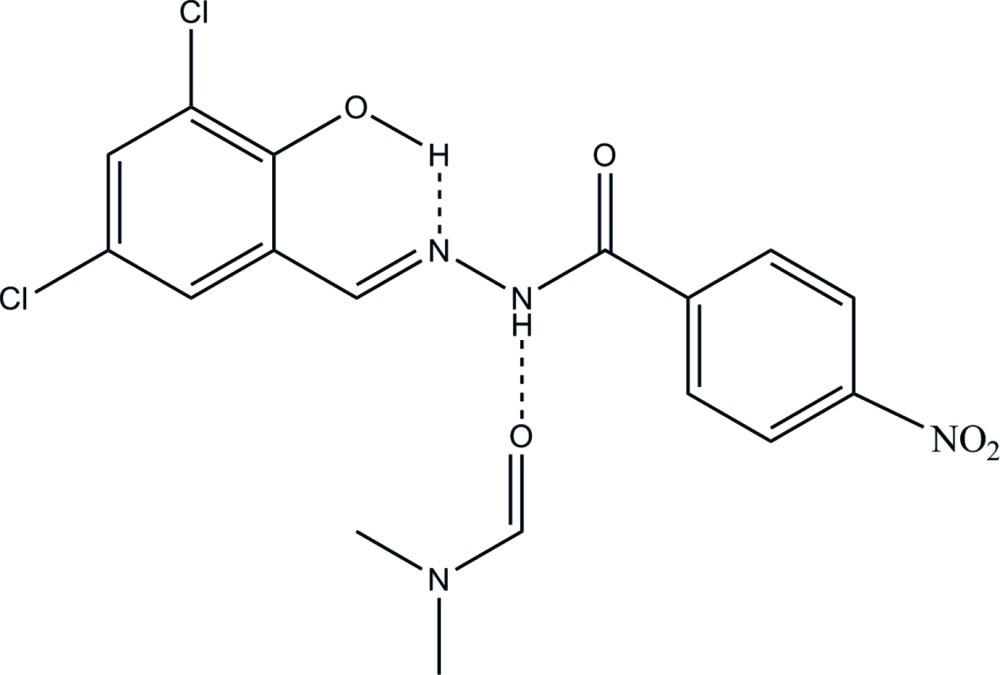


## Experimental   

### Crystal data  

C_14_H_9_Cl_2_N_3_O_4_·C_3_H_7_NO
*M* *_r_* = 427.24
Triclinic, 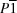

*a* = 7.8853 (6) Å
*b* = 11.9445 (10) Å
*c* = 11.9521 (15) Å
α = 114.408 (6)°
β = 102.895 (7)°
γ = 98.939 (5)°
*V* = 959.60 (17) Å^3^
*Z* = 2
Mo *K*α radiation
μ = 0.38 mm^−1^
*T* = 296 K
0.40 × 0.11 × 0.09 mm

### Data collection  

Bruker Kappa APEXII CCD Diffractometer
Absorption correction: multi-scan (*SADABS*; Bruker, 2004 ▸) *T*_min_ = 0.834, *T*_max_ = 0.929
7569 measured reflections
4660 independent reflections
3000 reflections with *I* > 2σ(*I*)
*R*_int_ = 0.019

### Refinement  

*R*[*F* ^2^ > 2σ(*F* ^2^)] = 0.043
*wR*(*F* ^2^) = 0.159
*S* = 0.95
4660 reflections
263 parameters
2 restraints
H atoms treated by a mixture of independent and constrained refinement
Δρ_max_ = 0.23 e Å^−3^
Δρ_min_ = −0.19 e Å^−3^

### 

Data collection: *APEX2* (Bruker, 2004 ▸); cell refinement: *APEX2* and *SAINT* (Bruker, 2004 ▸); data reduction: *SAINT* and *XPREP* (Bruker, 2004 ▸); program(s) used to solve structure: *SHELXS97* (Sheldrick, 2008 ▸); program(s) used to refine structure: *SHELXL2014* (Sheldrick, 2015 ▸); molecular graphics: *ORTEPIII* (Burnett & Johnson, 1996 ▸); software used to prepare material for publication: *publCIF* (Westrip, 2010 ▸).

## Supplementary Material

Crystal structure: contains datablock(s) I. DOI: 10.1107/S2056989015018290/yk2106sup1.cif

Structure factors: contains datablock(s) I. DOI: 10.1107/S2056989015018290/yk2106Isup2.hkl

Supporting information file. DOI: 10.1107/S2056989015018290/yk2106Isup3.cml

**Fig. 1.**
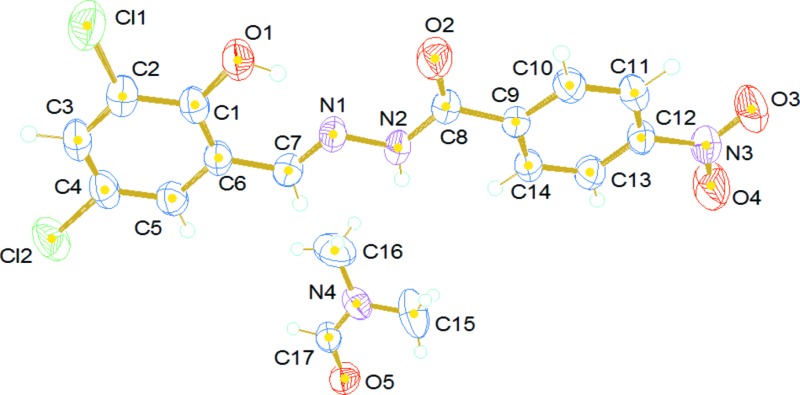
ORTEP view of the title compound, drawn with 50% probability displacement ellipsoids for the non-H atoms.

**Fig. 2.**
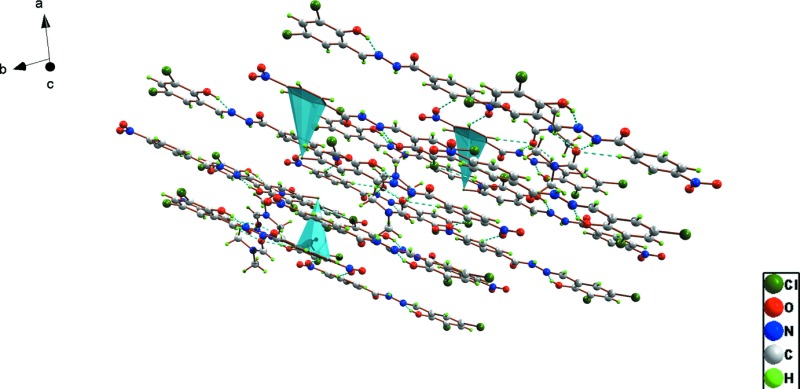
Diagram showing molecular packing viewed along the c axis along with intermolecular interactions.

ORTEP . DOI: 10.1107/S2056989015018290/yk2106fig1.tif
*ORTEP* view of the title compound, drawn with 50% probability displacement ellipsoids for the non-H atoms.

c . DOI: 10.1107/S2056989015018290/yk2106fig2.tif
Diagram showing mol­ecular packing viewed along the *c* axis along with inter­molecular inter­actions..

CCDC reference: 1428612

Additional supporting information:  crystallographic information; 3D view; checkCIF report

## Figures and Tables

**Table 1:** Hydrogen-bond geometry (, )

*D*H*A*	*D*H	H*A*	*D* *A*	*D*H*A*
O1H1N1	0.84(1)	1.82(2)	2.581(2)	151(3)
N2H2O5^i^	0.87(1)	1.90(1)	2.757(2)	169(3)
C3H3Cl1^ii^	0.93	2.92	3.836(2)	169
C7H7O5^i^	0.93	2.38	3.145(3)	139
C13H13O4^iii^	0.93	2.42	3.231(3)	146

Symmetry codes: (i) 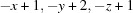; (ii) 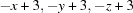; (iii) 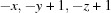.

## supplementary crystallographic information

### S1. Comment

Recent studies of hydrazones emphasis the importance of the hydrazone functional group in various fields ranging from organic synthesis and medicinal chemistry to supra­molecular chemistry (Su & Aprahamian, 2014). They have growing importance because of their biological applications (Nair *et al.*, 2014; Prasanna & Kumar, 2013; Hollo *et al.*, 2014). Here we discuss the synthesis of N'-[(E)-(3,5-di­chloro-2-hy­droxy­phenyl)­methyl­idene]-4-nitro­benzohydrazide di­methyl­formamide monosolvate from 3,5-di­chloro­salicyl­aldehyde and 4-nitro­benzoyl hydrazide. By this reaction, we obtained a novel di­methyl­formamide solvated aroylhydrazone in a simple condensation reaction.

The title compound, C_14_H_9_C_l2_N_3_O_4_·C_3_H_7_NO, adopts an *E* configuration with respect to C7═N1 bond (Fig. 1). The two aromatic rings of the molecule are almost in a plane with a slight twist with a dihedral angle of 8.96 (11) °. The C7═N1 and C8═O2 bond distances [1.269 (3) and 1.210 (2) Å, respectively] are very close to the formal C═N and C═O bond lengths. An intra­molecular hydrogen bond is found between N1 and the H atom of the phenolic group with a D···A distance of 2.581 (2) Å. Each hydrazone molecule forms one classical inter­molecular N—H···O hydrogen bond (to di­methyl­formamide molecule) and three non-classical C–H···O inter­molecular hydrogen bonds. The pairs of non-classical C13–H···O4 inter­actions with D···A distance of 3.232 (3) Å (Table 1) connect molecules into centrosymmetric dimers, and these dimers are connected by means of C–H···Cl inter­actions into chains along [3 2 2]. The packing diagram showing all hydrogen bonds and C—Cl···π inter­actions viewed along *c* axis is presented in Fig. 2.

### S2. Synthesis and crystallization

The title compound was prepared by adapting a reported procedure (Bessy *et al*, 2006) as described below. 3,5-Di­chloro­salicyl­aldehyde (0.191 g, 1 mmol) and 4-nitro­benzoyl hydrazide (0.181 g, 1 mmol) were dissolved in 10 mL of DMF. The solution was heated to boiling for 15 min, cooled to room temperature and then poured to 40 mL of water containing crushed ice and 1 mL of concentrated sulfuric acid. The pale yellow colored solid product was separated, washed with DMF and dried over P_4_O_10_*in vacuo*. Single crystals of the title compound suitable for X-ray analysis were obtained by recrystallization from di­methyl­formamide.

### S3. Refinement

All H atoms on C were placed in calculated positions, guided by difference map, with C—H bond distances of 0.93-0.96 Å. H atoms were assigned *U*_iso_(H) values of 1.2Ueq(carrier). H atoms attached to N2 and O1 were located from a difference Fourier map and the bond distances are restrained to 0.88±0.01 and 0.84±0.01 Å, respectively. The reflections (0 0 1), (0 -1 1) and (0 1 0) were omitted owing to bad agreement.

### Crystal data

**Table:**

C_14_H_9_Cl_2_N_3_O_4_·C_3_H_7_NO	*Z* = 2
*M**_r_* = 427.24	*F*(000) = 440
Triclinic, *P*1	*D*_x_ = 1.479 Mg m^−^^3^
*a* = 7.8853 (6) Å	Mo *K*α radiation, λ = 0.71073 Å
*b* = 11.9445 (10) Å	Cell parameters from 2537 reflections
*c* = 11.9521 (15) Å	θ = 2.8–28.1°
α = 114.408 (6)°	µ = 0.38 mm^−^^1^
β = 102.895 (7)°	*T* = 296 K
γ = 98.939 (5)°	Needle, pale yellow
*V* = 959.60 (17) Å^3^	0.40 × 0.11 × 0.09 mm

### Data collection

**Table:**

Bruker Kappa APEXII CCD Diffractometer	3000 reflections with *I* > 2σ(*I*)
Radiation source: fine-focus sealed tube	*R*_int_ = 0.019
ω and φ scan	θ_max_ = 28.4°, θ_min_ = 2.8°
Absorption correction: multi-scan (*SADABS*; Bruker, 2004)	*h* = −10→9
*T*_min_ = 0.834, *T*_max_ = 0.929	*k* = −15→15
7569 measured reflections	*l* = −15→15
4660 independent reflections	

### Refinement

**Table:**

Refinement on *F*^2^	2 restraints
Least-squares matrix: full	Hydrogen site location: mixed
*R*[*F*^2^ > 2σ(*F*^2^)] = 0.043	H atoms treated by a mixture of independent and constrained refinement
*wR*(*F*^2^) = 0.159	*w* = 1/[σ^2^(*F*_o_^2^) + (0.0998*P*)^2^ + 0.0446*P*] where *P* = (*F*_o_^2^ + 2*F*_c_^2^)/3
*S* = 0.95	(Δ/σ)_max_ = 0.001
4660 reflections	Δρ_max_ = 0.23 e Å^−^^3^
263 parameters	Δρ_min_ = −0.19 e Å^−^^3^

### Special details

**Table:**

Geometry. All e.s.d.'s (except the e.s.d. in the dihedral angle between two l.s. planes) are estimated using the full covariance matrix. The cell e.s.d.'s are taken into account individually in the estimation of e.s.d.'s in distances, angles and torsion angles; correlations between e.s.d.'s in cell parameters are only used when they are defined by crystal symmetry. An approximate (isotropic) treatment of cell e.s.d.'s is used for estimating e.s.d.'s involving l.s. planes.

### Fractional atomic coordinates and isotropic or equivalent isotropic displacement parameters (Å^2^)

**Table:**

	*x*	*y*	*z*	*U*_iso_*/*U*_eq_	
Cl1	1.35723 (9)	1.29047 (6)	1.47197 (6)	0.0756 (2)	
Cl2	1.23273 (9)	1.51632 (6)	1.17091 (7)	0.0756 (2)	
O1	1.0508 (2)	1.08266 (15)	1.27329 (15)	0.0576 (4)	
O2	0.6756 (2)	0.77509 (15)	1.08867 (15)	0.0652 (4)	
O3	−0.0311 (3)	0.25861 (16)	0.6472 (2)	0.0864 (6)	
O4	−0.0770 (3)	0.3439 (2)	0.5227 (2)	0.0989 (7)	
O5	0.4796 (2)	1.06202 (17)	0.22900 (16)	0.0668 (4)	
N1	0.78454 (19)	0.97794 (15)	1.05922 (16)	0.0457 (4)	
N2	0.6378 (2)	0.87905 (15)	0.96978 (16)	0.0454 (4)	
N3	0.0045 (2)	0.34550 (18)	0.6219 (2)	0.0619 (5)	
N4	0.5894 (3)	0.96448 (18)	0.34168 (18)	0.0616 (5)	
C1	1.0866 (2)	1.18037 (18)	1.24598 (19)	0.0441 (4)	
C2	1.2306 (3)	1.2870 (2)	1.3331 (2)	0.0512 (5)	
C3	1.2741 (3)	1.39056 (18)	1.3111 (2)	0.0529 (5)	
H3	1.3694	1.4622	1.3714	0.063*	
C4	1.1756 (3)	1.38669 (19)	1.1995 (2)	0.0521 (5)	
C5	1.0326 (2)	1.28330 (19)	1.1101 (2)	0.0489 (5)	
H5	0.9675	1.2824	1.0344	0.059*	
C6	0.9853 (2)	1.17920 (17)	1.13350 (19)	0.0426 (4)	
C7	0.8316 (2)	1.07285 (19)	1.03936 (19)	0.0464 (4)	
H7	0.7673	1.0738	0.9645	0.056*	
C8	0.5925 (2)	0.77882 (18)	0.99269 (18)	0.0428 (4)	
C9	0.4325 (2)	0.67029 (17)	0.89195 (18)	0.0397 (4)	
C10	0.3727 (3)	0.57541 (19)	0.9227 (2)	0.0496 (5)	
H10	0.4276	0.5841	1.0044	0.060*	
C11	0.2333 (3)	0.4681 (2)	0.8348 (2)	0.0548 (5)	
H11	0.1944	0.4034	0.8551	0.066*	
C12	0.1536 (2)	0.45939 (18)	0.7164 (2)	0.0470 (4)	
C13	0.2077 (3)	0.5529 (2)	0.6837 (2)	0.0507 (5)	
H13	0.1497	0.5449	0.6029	0.061*	
C14	0.3485 (2)	0.65882 (19)	0.77184 (19)	0.0456 (4)	
H14	0.3873	0.7228	0.7506	0.055*	
C15	0.4196 (5)	0.8698 (3)	0.2961 (4)	0.1047 (11)	
H15A	0.3223	0.9022	0.2699	0.157*	
H15B	0.4052	0.8506	0.3645	0.157*	
H15C	0.4177	0.7934	0.2236	0.157*	
C16	0.7510 (5)	0.9501 (3)	0.4148 (3)	0.0998 (10)	
H16A	0.7692	0.8691	0.3641	0.150*	
H16B	0.7365	0.9538	0.4938	0.150*	
H16C	0.8541	1.0179	0.4349	0.150*	
C17	0.6030 (3)	1.0510 (2)	0.3016 (2)	0.0531 (5)	
H17	0.7162	1.1087	0.3310	0.064*	
H2	0.589 (3)	0.888 (2)	0.9019 (17)	0.072 (8)*	
H1	0.962 (3)	1.028 (2)	1.2106 (19)	0.090 (9)*	

### Atomic displacement parameters (Å^2^)

**Table:**

	*U*^11^	*U*^22^	*U*^33^	*U*^12^	*U*^13^	*U*^23^
Cl1	0.0735 (4)	0.0650 (4)	0.0560 (3)	−0.0053 (3)	−0.0032 (3)	0.0191 (3)
Cl2	0.0772 (4)	0.0489 (3)	0.1074 (5)	0.0066 (3)	0.0394 (4)	0.0413 (4)
O1	0.0540 (8)	0.0471 (9)	0.0590 (9)	−0.0035 (7)	0.0066 (7)	0.0249 (8)
O2	0.0644 (9)	0.0599 (10)	0.0530 (9)	−0.0038 (7)	−0.0047 (7)	0.0286 (8)
O3	0.0830 (12)	0.0449 (10)	0.1029 (15)	−0.0120 (8)	0.0080 (11)	0.0295 (10)
O4	0.0887 (13)	0.0704 (12)	0.0839 (13)	−0.0243 (10)	−0.0282 (10)	0.0310 (11)
O5	0.0629 (9)	0.0687 (11)	0.0651 (10)	0.0103 (8)	0.0063 (8)	0.0375 (9)
N1	0.0368 (8)	0.0370 (8)	0.0492 (9)	0.0012 (6)	0.0089 (7)	0.0128 (7)
N2	0.0367 (8)	0.0378 (8)	0.0467 (9)	−0.0013 (6)	0.0027 (7)	0.0152 (8)
N3	0.0514 (10)	0.0408 (10)	0.0705 (13)	−0.0025 (8)	0.0058 (9)	0.0171 (9)
N4	0.0783 (12)	0.0453 (10)	0.0564 (11)	0.0101 (9)	0.0138 (9)	0.0257 (9)
C1	0.0418 (9)	0.0370 (10)	0.0489 (11)	0.0042 (7)	0.0173 (8)	0.0169 (9)
C2	0.0449 (10)	0.0446 (11)	0.0487 (11)	0.0018 (8)	0.0140 (9)	0.0119 (9)
C3	0.0440 (10)	0.0368 (11)	0.0579 (12)	−0.0015 (8)	0.0172 (9)	0.0078 (10)
C4	0.0468 (10)	0.0365 (10)	0.0702 (14)	0.0055 (8)	0.0282 (10)	0.0197 (10)
C5	0.0441 (10)	0.0433 (11)	0.0584 (12)	0.0096 (8)	0.0187 (9)	0.0228 (10)
C6	0.0363 (8)	0.0342 (9)	0.0487 (10)	0.0052 (7)	0.0155 (8)	0.0122 (8)
C7	0.0389 (9)	0.0423 (11)	0.0495 (11)	0.0074 (8)	0.0094 (8)	0.0174 (9)
C8	0.0372 (9)	0.0391 (10)	0.0424 (10)	0.0035 (7)	0.0080 (7)	0.0150 (8)
C9	0.0349 (8)	0.0351 (9)	0.0441 (9)	0.0061 (7)	0.0098 (7)	0.0165 (8)
C10	0.0497 (10)	0.0467 (11)	0.0499 (11)	0.0057 (8)	0.0082 (9)	0.0267 (10)
C11	0.0526 (11)	0.0412 (11)	0.0673 (13)	0.0016 (8)	0.0121 (10)	0.0298 (11)
C12	0.0399 (9)	0.0345 (10)	0.0540 (11)	0.0019 (7)	0.0092 (8)	0.0149 (9)
C13	0.0478 (10)	0.0463 (11)	0.0484 (11)	0.0042 (8)	0.0053 (9)	0.0214 (9)
C14	0.0424 (9)	0.0399 (10)	0.0507 (11)	0.0031 (8)	0.0093 (8)	0.0232 (9)
C15	0.121 (3)	0.072 (2)	0.111 (2)	−0.0127 (17)	0.039 (2)	0.0468 (19)
C16	0.128 (3)	0.086 (2)	0.0810 (19)	0.041 (2)	0.0054 (18)	0.0460 (18)
C17	0.0537 (11)	0.0449 (11)	0.0524 (12)	0.0040 (9)	0.0108 (9)	0.0216 (10)

### Geometric parameters (Å, º)

**Table:**

Cl1—C2	1.716 (2)	C5—C6	1.397 (3)
Cl2—C4	1.734 (2)	C5—H5	0.9300
O1—C1	1.342 (2)	C6—C7	1.442 (3)
O1—H1	0.835 (10)	C7—H7	0.9300
O2—C8	1.210 (2)	C8—C9	1.498 (2)
O3—N3	1.206 (2)	C9—C10	1.378 (3)
O4—N3	1.207 (3)	C9—C14	1.380 (3)
O5—C17	1.214 (2)	C10—C11	1.375 (3)
N1—C7	1.269 (2)	C10—H10	0.9300
N1—N2	1.363 (2)	C11—C12	1.367 (3)
N2—C8	1.348 (2)	C11—H11	0.9300
N2—H2	0.872 (10)	C12—C13	1.367 (3)
N3—C12	1.466 (3)	C13—C14	1.372 (3)
N4—C17	1.306 (3)	C13—H13	0.9300
N4—C15	1.437 (3)	C14—H14	0.9300
N4—C16	1.454 (3)	C15—H15A	0.9600
C1—C2	1.386 (3)	C15—H15B	0.9600
C1—C6	1.396 (3)	C15—H15C	0.9600
C2—C3	1.376 (3)	C16—H16A	0.9600
C3—C4	1.363 (3)	C16—H16B	0.9600
C3—H3	0.9300	C16—H16C	0.9600
C4—C5	1.370 (3)	C17—H17	0.9300
			
C1—O1—H1	105 (2)	N2—C8—C9	116.40 (16)
C7—N1—N2	118.71 (16)	C10—C9—C14	119.48 (17)
C8—N2—N1	117.36 (15)	C10—C9—C8	116.28 (16)
C8—N2—H2	128.4 (18)	C14—C9—C8	124.20 (16)
N1—N2—H2	114.1 (18)	C11—C10—C9	121.12 (18)
O3—N3—O4	123.3 (2)	C11—C10—H10	119.4
O3—N3—C12	118.6 (2)	C9—C10—H10	119.4
O4—N3—C12	118.07 (19)	C12—C11—C10	117.92 (18)
C17—N4—C15	120.0 (2)	C12—C11—H11	121.0
C17—N4—C16	120.2 (2)	C10—C11—H11	121.0
C15—N4—C16	119.0 (2)	C13—C12—C11	122.36 (18)
O1—C1—C2	118.66 (18)	C13—C12—N3	119.26 (19)
O1—C1—C6	122.82 (16)	C11—C12—N3	118.38 (18)
C2—C1—C6	118.52 (17)	C12—C13—C14	119.15 (18)
C3—C2—C1	121.57 (19)	C12—C13—H13	120.4
C3—C2—Cl1	119.12 (16)	C14—C13—H13	120.4
C1—C2—Cl1	119.31 (16)	C13—C14—C9	119.95 (17)
C4—C3—C2	119.06 (18)	C13—C14—H14	120.0
C4—C3—H3	120.5	C9—C14—H14	120.0
C2—C3—H3	120.5	N4—C15—H15A	109.5
C3—C4—C5	121.61 (19)	N4—C15—H15B	109.5
C3—C4—Cl2	119.00 (16)	H15A—C15—H15B	109.5
C5—C4—Cl2	119.39 (18)	N4—C15—H15C	109.5
C4—C5—C6	119.5 (2)	H15A—C15—H15C	109.5
C4—C5—H5	120.2	H15B—C15—H15C	109.5
C6—C5—H5	120.2	N4—C16—H16A	109.5
C1—C6—C5	119.70 (17)	N4—C16—H16B	109.5
C1—C6—C7	121.85 (16)	H16A—C16—H16B	109.5
C5—C6—C7	118.44 (18)	N4—C16—H16C	109.5
N1—C7—C6	119.61 (18)	H16A—C16—H16C	109.5
N1—C7—H7	120.2	H16B—C16—H16C	109.5
C6—C7—H7	120.2	O5—C17—N4	125.3 (2)
O2—C8—N2	122.52 (17)	O5—C17—H17	117.4
O2—C8—C9	121.07 (17)	N4—C17—H17	117.4
			
C7—N1—N2—C8	179.15 (17)	N1—N2—C8—C9	−179.03 (15)
O1—C1—C2—C3	−179.80 (17)	O2—C8—C9—C10	8.0 (3)
C6—C1—C2—C3	0.3 (3)	N2—C8—C9—C10	−172.24 (16)
O1—C1—C2—Cl1	0.0 (3)	O2—C8—C9—C14	−169.75 (19)
C6—C1—C2—Cl1	−179.94 (14)	N2—C8—C9—C14	10.0 (3)
C1—C2—C3—C4	−1.5 (3)	C14—C9—C10—C11	1.4 (3)
Cl1—C2—C3—C4	178.75 (15)	C8—C9—C10—C11	−176.44 (18)
C2—C3—C4—C5	1.2 (3)	C9—C10—C11—C12	−1.1 (3)
C2—C3—C4—Cl2	−179.09 (15)	C10—C11—C12—C13	−0.1 (3)
C3—C4—C5—C6	0.3 (3)	C10—C11—C12—N3	−179.89 (18)
Cl2—C4—C5—C6	−179.43 (13)	O3—N3—C12—C13	173.37 (19)
O1—C1—C6—C5	−178.71 (16)	O4—N3—C12—C13	−7.0 (3)
C2—C1—C6—C5	1.2 (3)	O3—N3—C12—C11	−6.8 (3)
O1—C1—C6—C7	1.2 (3)	O4—N3—C12—C11	172.8 (2)
C2—C1—C6—C7	−178.87 (18)	C11—C12—C13—C14	0.9 (3)
C4—C5—C6—C1	−1.5 (3)	N3—C12—C13—C14	−179.30 (18)
C4—C5—C6—C7	178.56 (17)	C12—C13—C14—C9	−0.5 (3)
N2—N1—C7—C6	179.29 (15)	C10—C9—C14—C13	−0.6 (3)
C1—C6—C7—N1	1.0 (3)	C8—C9—C14—C13	177.11 (18)
C5—C6—C7—N1	−179.11 (16)	C15—N4—C17—O5	2.2 (4)
N1—N2—C8—O2	0.7 (3)	C16—N4—C17—O5	172.0 (2)

### Hydrogen-bond geometry (Å, º)

**Table:**

*D*—H···*A*	*D*—H	H···*A*	*D*···*A*	*D*—H···*A*
N2—H2···O5^i^	0.87 (1)	1.90 (1)	2.757 (2)	169 (3)
C3—H3···Cl1^ii^	0.93	2.92	3.836 (2)	169
C7—H7···O5^i^	0.93	2.38	3.145 (3)	139
C13—H13···O4^iii^	0.93	2.42	3.231 (3)	146
O1—H1···N1	0.84 (1)	1.82 (2)	2.581 (2)	151 (3)

Symmetry codes: (i) −*x*+1, −*y*+2, −*z*+1; (ii) −*x*+3, −*y*+3, −*z*+3; (iii) −*x*, −*y*+1, −*z*+1.
