# Supplementary figures and images for: Crystal structure of N′-[(E)-3,5-di­chloro-2-hy­droxy­benzyl­idene]-4-nitro­benzo­hydrazide di­methyl­formamide monosolvate
Source: Acta Crystallogr E Crystallogr Commun. 2015 Oct 7;71(Pt 11):o826–7. doi: 10.1107/S2056989015018290 (PMC4645068; doi:10.1107/S2056989015018290)

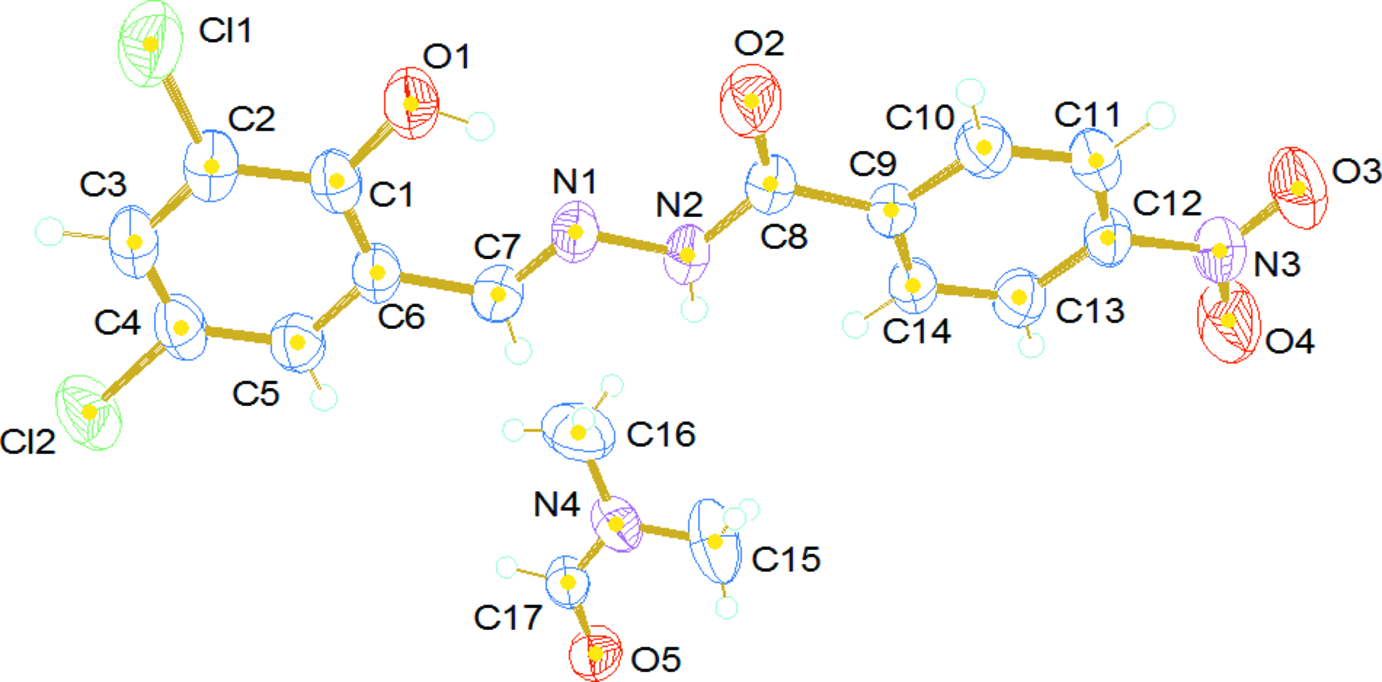

Supplement: Supplementary file 4 [file e-71-0o826-fig1.tif]

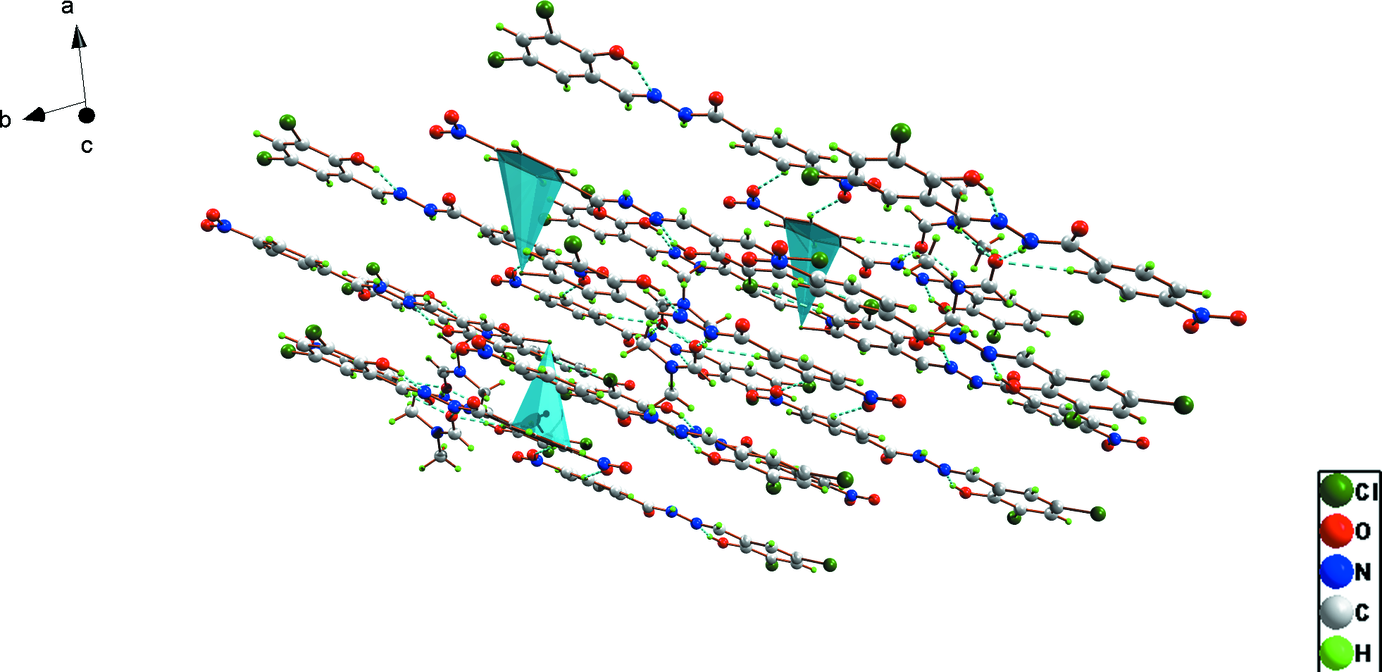

Supplement: Supplementary file 5 [file e-71-0o826-fig2.tif]
